# Multimodality Imaging-Guided Aspiration of a Right Ventricular Mass – Cardiac Metastasis Unmasked: A Case Report

**DOI:** 10.1016/j.jscai.2026.105337

**Published:** 2026-04-30

**Authors:** Ahmad Al-Sahli, Anas Fares, Stephanie Younes, Sourabh Prabhakar, Rachel K. Trouten, Ahmad Younes

**Affiliations:** a Jobst Vascular Institute, ProMedica Toledo Hospital, Toledo, Ohio; b Division of Cardiovascular Medicine, University of Toledo, Toledo, Ohio; c ProMedica Heart Institute, ProMedica Toledo Hospital, Toledo, Ohio; d Division of Cardiothoracic Surgery, University of Washington Medical Center, Seattle, Washington

**Keywords:** AngioVac, case report, debulking, intracardiac echocardiogram, right ventricular mass

## Abstract

This case describes the safe and effective percutaneous removal of a large right ventricular mass using the AngioVac system (AngioDynamics) guided by multimodality imaging. Intracardiac echocardiography was used throughout the procedure for real-time navigation and mass engagement in conjunction with fluoroscopy. Cardiac magnetic resonance imaging and positron emission tomography-computed tomography were performed after surgery to further characterize the mass and confirm metastatic involvement. Histopathology revealed metastatic squamous cell carcinoma, underscoring the diagnostic and therapeutic value of comprehensive imaging integration.

## Case Report

**Figure 1:**
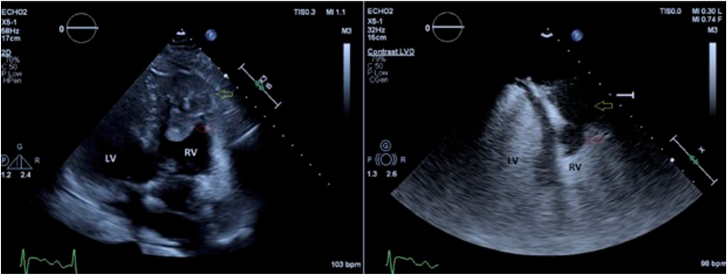
**Preprocedural transthoracic echocardiographic imaging.** (Left) Apical 4-chamber view demonstrating a large, well-demarcated, echodense mass involving the right ventricle (RV) lateral wall and apex (yellow arrow), with an adjacent, more homogeneous echogenic thrombus (red arrow) in the RV apex. (Right) Contrast-enhanced apical 4-chamber view improves delineation of both the RV mass (yellow arrow) and the associated thrombus (red arrow), confirming their intracavitary extension.

A 62-year-old man with a history of metastatic squamous cell carcinoma of the parotid gland presented with syncope. Initial vitals significant for tachycardia with a heart rate of 113 beats per minute, requiring 2L supplemental oxygen. Evaluation revealed bilateral pulmonary emboli. Transthoracic echocardiography identified a large right ventricular mass measuring 4.3 × 2.9 × 3.1 cm with a mobile component (Fig 1) (Supplemental Video 1).

Given its uncertain etiology, location on imaging, and potential embolic risk, multidisciplinary discussions among the heart team, including interventional cardiology, cardiac imaging specialists, and cardiac surgery with careful review of the patient’s echocardiogram and computed tomography angiography of the chest. Given the patient presentation and respiratory status, percutaneous debulking of the right ventricle mobile mass was recommended over anticoagulation alone. Consensus was made to proceed with percutaneous aspiration using the AngioVac system (AngioDynamics) under combined fluoroscopic and intracardiac echocardiography guidance with moderate sedation, avoiding general anesthesia if possible. Intracardiac echocardiography guidance was selected for its superior near-field visualization of right-sided cardiac structures and real-time intraprocedural imaging without the need for general anesthesia and has had a growing role in structural heart procedures,^1^ without an associated increased risk of systemic embolism compared with transesophageal echocardiography.^2^

**Figure 2:**
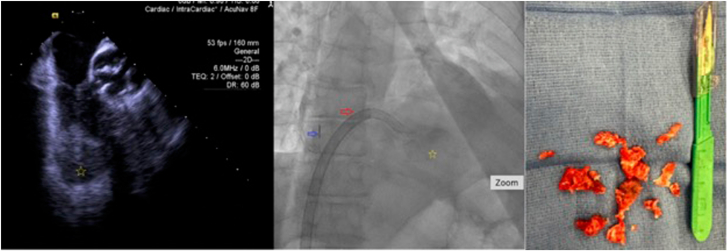
**Procedural imaging guidance and gross tissue specimen.** (Left) Intracardiac echocardiography (ICE) image showing a right ventricular mass with broad-based attachment to the lateral wall and extension toward the apex. ICE was used throughout the procedure for its superior visualization of right-sided structures, facilitating cannula positioning, confirming mass engagement, and continuously monitoring proximity to valves to avoid injury. (Center) Fluoroscopic image demonstrating the AngioVac cannula (red arrow) advanced into the right ventricule via right femoral venous access. ICE (blue arrow), positioned in the right atrium, provided real-time imaging to guide the curved aspiration catheter and assist in engaging the mass and associated thrombus (yellow star). (Right) Gross specimen aspirated via the AngioVac circuit, revealing multiple fragments submitted for histopathology. Final analysis showed organizing thrombus admixed with p40-positive atypical cells consistent with metastatic squamous cell carcinoma.

The procedure was performed via right femoral venous access using a 26F GORE DRYSEAL sheath (W. L. Gore & Associates) placed at the inferior vena cava/right atrial junction and a 17F return cannula in the left femoral vein. Secondary access was obtained in the right common femoral vein with a 9F 35-cm sheath advanced into the inferior vena cava for intracardiac echocardiography catheter placement. Intracardiac echocardiography provided real-time near-field imaging, guiding precise advancement of the AngioVac cannula toward the right ventricular mass and assisting in valve preservation (Supplemental Video 2). Versacore wire was advanced carefully to the right pulmonary artery guided by a 5F Judkins right catheter. Wire was exchanged for an Amplatz wire for additional support. The trajectory of the AngioVac cannula was optimized by tracking over the Amplatz wire, which was then pulled. A combination of fluoroscopy and intracardiac echocardiography guidance was used to avoid trauma to the tricuspid and pulmonic valves. Flow was initiated at 3 to 4 L/min through the extracorporeal circuit, and aspirated material was sent for histopathologic analysis (Fig. 2) (Supplemental Videos 3 and 4). Postprocedural course was uncomplicated, bilateral groin access was managed with figure-of-8 stitches, and anticoagulation was never interrupted. The patient was weaned off supplemental oxygen and eventually discharged on oral anticoagulation.

**Figure 3:**
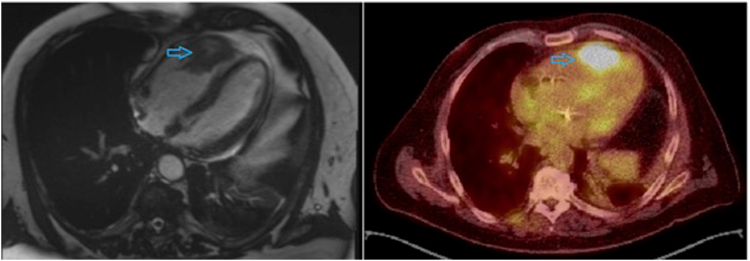
**Postprocedural multimodality imaging.** (Left) Cardiac magnetic resonance imaging showing a heterogeneous, solid, and partially cystic mass (blue arrow) involving the right ventricular lateral wall, extending to the apex with papillary muscle involvement. The mass exhibits postcontrast enhancement and central T2 heterogeneity suggestive of necrosis. These findings are atypical for thrombus and raise concern for metastatic disease. (Right) Positron emission tomography-computed tomography shows marked luorodeoxyglucose uptake within the corresponding right ventricular region (blue arrow), with a maximum standardized uptake value of 15.37, consistent with high metabolic activity and further supporting the diagnosis of malignancy. Additional findings included a hypermetabolic pretracheal lymph node and a posterior neck cutaneous focus, indicating further sites of concern. Multimodal imaging collectively supported the diagnosis of intracardiac metastasis.

Although tissue was obtained for pathology, given the echocardiographic appearance, a cardiac magnetic resonance imaging (MRI) was subsequently performed to characterize the residual mass and inform oncologic planning while awaiting histopathologic results. The MRI revealed a heterogeneous, contrast-enhancing right ventricular mass with central necrosis. Positron emission tomography-computed tomography confirmed intense radiotracer, fluorodeoxyglucose uptake (standardized uptake value max, 15.37) (Fig. 3). Pathology revealed organizing thrombus with p40-positive atypical cells consistent with metastatic squamous cell carcinoma. The patient was referred for stereotactic body radiation therapy and future systemic therapy.

Previous reports have described the feasibility of AngioVac aspiration for right-sided cardiac masses and emphasized the value of multimodality imaging in their evaluation.^3^, ^4^, ^5^ In our case, intracardiac echocardiography was favored over transesophageal echocardiography because of its superior near-field imaging of right-sided structures, avoidance of general anesthesia, lower procedural turnover times, and potential reduction in hospital resource utilization. Combined with cardiac MRI and positron emission tomography-computed tomography, this approach enabled safe percutaneous mass removal, definitive diagnosis of metastatic squamous cell carcinoma, and informed oncologic management.

## CRediT authorship contribution statement

**Ahmad Al-Sahli:** Writing – original draft, Writing – review & editing. **Anas Fares:** Writing – original draft. **Stephanie Younes:** Supervision, Writing – review & editing. **Sourabh Prabhakar:** Writing – original draft. **Rachel K. Trouten:** Writing – original draft. **Ahmad Younes:** Supervision, Writing – original draft, Writing – review & editing.

## Supplementary material

Supplemental Video 1 **Transthoracic** echo video. Apical 4-chamber video demonstrating a large mobile, well-demarcated, echodense mass involving the right ventricle.

Supplemental Video 2 Predebulking. Video of intracardiac echo demonstrating a mass in the right ventricle with a mobile component.

Supplemental Video 3 **AngioVac** of right ventricle 2. Video demonstrating intracardiac echo-guided AngioVac engaging the right ventricular mass.

Supplemental Video 4 Postdebulking. Video demonstrating intracardiac echo of RV mass post debulking. RV, right ventricle.

## Footnotes

To access the supplementary material accompanying this article, visit the online version of the *Journal of the Society for Cardiovascular Angiography & Interventions* at 10.1016/j.jscai.2026.105337.

## References

[1] Eleid M.F., Chung C.J., Daniels M.J. (2024). SCAI position statement on intracardiac echocardiography to guide structural heart disease interventions. J Soc Cardiovasc Angiogr Interv.

[2] Hu X., Jiang W., Wang X., ICE vs TEE Study Investigators (2025). Intracardiac vs transesophageal echocardiography in atrial fibrillation ablation: a randomized clinical trial. JAMA Cardiol.

[3] Madan N, von Buchwald CL, Golemi L, Iskander M, Attanasio S (2023). Aspiration of right sided intracardiac masses in high-risk surgical patients using AngioVac: a case series and review of literature. Cardiovasc Revasc Med.

[4] Motwani M., Kidambi A., Herzog B.A., Uddin A., Greenwood J.P., Plein S. (2013). MR imaging of cardiac tumors and masses: a review of methods and clinical applications. Radiology.

[5] Bhattacharyya S., Khattar R.S., Gujral D.M., Senior R. (2014). Cardiac tumors: the role of cardiovascular imaging. Expert Rev Cardiovasc Ther.

